# Joint associations of physical activity and sedentary time with adiposity during adolescence: ALSPAC

**DOI:** 10.1093/eurpub/ckac023

**Published:** 2022-04-13

**Authors:** Soyang Kwon, Ulf Ekelund, Namratha R Kandula, Kathleen F Janz

**Affiliations:** Department of Pediatrics, Northwestern University, Chicago, IL, USA; Department of Sports Medicine, Norwegian School of Sports Sciences, Oslo, Norway; Departments of Medicine and Preventive Medicine, Northwestern University, Chicago, IL, USA; Department of Health and Human Physiology, University of Iowa, Iowa City, IA, USA

## Abstract

**Background:**

In developing evidence-based physical activity (PA) guidelines for youth, a knowledge gap exists regarding the health effects of sedentary time (SED). The aim of this study was to determine the joint associations of moderate- and vigorous-intensity PA (MVPA) and SED with adiposity during adolescence.

**Methods:**

The study sample was 2619 non-obese participants (56.7% female) from the UK Avon Longitudinal Study of Parents and Children. Accelerometer-measured MVPA and SED at age 11, 13, 15 years and self-reported TV viewing at age 13 and 16 years were used to create two exposure variables: six MVPA&SED combinations based on two MVPA patterns [≥60 (active) and <60 min/day (inactive)] and three SED patterns [≈25 (low), ≈30 (middle) and ≈35 min/h (high)] and six MVPA&TV combinations based on two MVPA patterns and three TV viewing patterns [<1–2 (low), 1–2 (middle) and >1–2 h/day (high)]. Adiposity was evaluated using fat mass index (FMI) at age 17 years.

**Results:**

SED was not significantly associated with FMI in either active or inactive adolescents. However, higher TV viewing was associated with higher FMI in both active [adjusted FMI = 4.53 vs. 5.09 (95% CI = 4.87, 5.33) for low TV vs. high TV] and inactive adolescents [adjusted FMI = 4.91 vs. 5.21 (95% CI = 5.02, 5.39) for low TV vs. high TV].

**Conclusions:**

Higher TV viewing time, but not total SED, was prospectively associated with higher adiposity among both active and inactive adolescents, suggesting a specific sedentary behavior target for public health.

## Introduction

The health benefits of physical activity (PA) are well recognized. A review^1^ for the 2018 Physical Activity Guidelines for Americans (PAG) summarized moderate to strong evidence that higher PA is associated with more favorable health status, including adiposity and cardiometabolic risk factors (e.g. plasma triglycerides and insulin), in youth. With the understanding that being physically active is not the opposite of being sedentary,^2^^,^^3^ sedentary time (SED) has received great attention as a potential independent risk factor for these health outcomes. The 2018 PAG review^1^ concluded that there is strong evidence to support the harmful effects of SED on Type 2 diabetes, cardiovascular disease and mortality in adults.^1^^,^^4–7^ The report additionally summarized that the relationship between SED and these health outcomes varies by moderate- and vigorous-intensity PA (MVPA) levels, suggesting that the harmful effects of SED could be attenuated by high PA. However, as noted in reviews for the 2018 PAG(1) and for the 2020 World Health Organization guidelines,^8^ there is insufficient evidence to determine if SED is related to poorer health outcomes in youth. Nonetheless, both reviews^1^^,^^8^ recognized that specific types of sedentary behavior might have different health effects. For example, the evidence is somewhat stronger for television (TV) viewing time than for total SED in youth.^1^^,^^8^

In youth, studies often examined SED after statistically adjusting for MVPA to determine the independent association with health outcomes and reported mixed results (no or positive association).^9–13^ It is unclear whether the link between SED and cardiometabolic health shown in some studies is the result of the direct effects of SED or an observation in relation to PA effects^8^ (e.g. displacement of PA or modification by PA). These potential pathways can be better understood by investigating the joint associations of PA and SED; however, only a few studies have investigated the joint associations of PA and SED in youth. For example, a meta-analysis by Ekelund et al.^14^ examined the cross-sectional joint associations of accelerometer-measured MVPA and SED with cardiometabolic risk factors and reported a clear and consistent association with MVPA in combinations with any SED levels, but no association with SED. Mielke et al.^15^ examined prospective joint associations of PA and SED in Brazilian adolescents and reported no associations for self-reported MVPA or SED at age 11 or 15 years with adiposity or cardiometabolic risk factors at age 18 years. Prospective investigations for the joint associations of accelerometer-measured PA and SED are lacking in youth. To address this knowledge gap, we examined the joint associations of accelerometer-measured PA and SED during adolescence with adiposity and cardiovascular risk factors at age 17 years.

## Methods

### Participants

**Figure 1:**
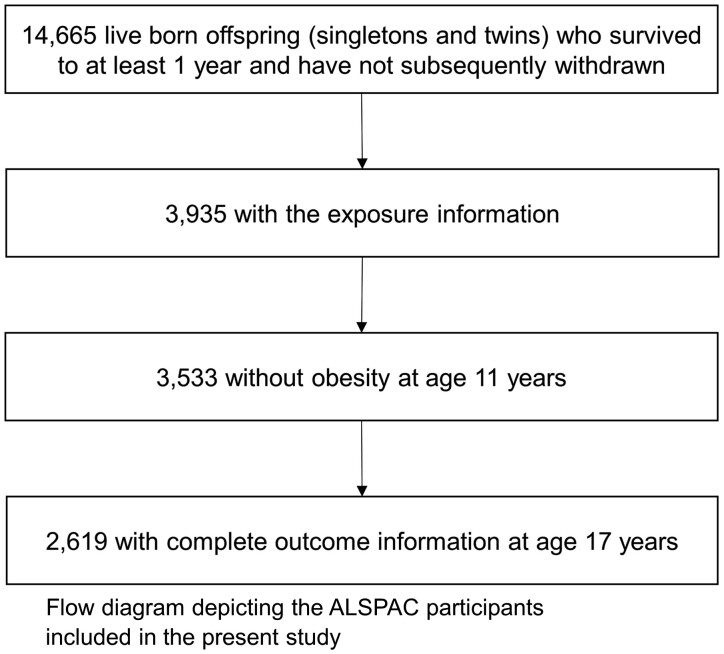
Flow diagram depicting the ALSPAC participants included in the present study.

We conducted a secondary analysis using data from the UK Avon Longitudinal Study of Parents and Children (ALSPAC). ALSPAC invited pregnant women resident in Avon, UK with expected dates of delivery 1 April 1991 to 31 December 1992 to take part in the study, resulting in 14 541 initial pregnancies enrolled. When the oldest children were ∼7 years of age, an attempt was made to bolster the initial sample with eligible cases who had failed to join the study originally. The total sample size for analyses using any data collected after the age of 7 is therefore 15 454 pregnancies, resulting in 15 589 fetuses. Of these, 14 901 were alive at 1 year of age. The study website contains details of all the data that is available through a fully searchable data dictionary and variable search tool (http://www.bristol.ac.uk/alspac/researchers/our-data/). In this report, we defined age 11 years as the baseline and age 17 years as the endpoint to focus on the adolescent period. This secondary analysis included data from a subset of the ALSPAC sample who were not obese at baseline, completed at least two ActiGraph accelerometer assessments between the baseline and the endpoint, and completed a dual-energy X-ray absorptiometry (DXA) scan at the endpoint (figure 1). Obesity at baseline was defined as the sex- and age-specific BMI percentile ≥95th percentile based on the 1990 British Growth Reference Charts. Informed consent for the use of data collected via questionnaires and clinics was obtained from participants following the recommendations of the ALSPAC Ethics and Law Committee at the time. Ethical approval for the ALSPAC was obtained from the ALSPAC Ethics and Law Committee and the Local Research Ethics Committees.

### Exposure measurements

The exposures of this study were MVPA and SED (total SED and TV viewing) between age 11 and 15 years. During research clinic visits at ages 11, 13 and 15 years (between 2003 and 2008), participants were given an ActiGraph accelerometer (Model 7164 at ages 11 and 13 years and GT1M at age 15 years; 6108 participants at age 11 years, 4785 at age 13 years and 2948 at age 15 years) and asked to wear the monitor over the hip during waking hours for 7 days. Participants were advised to take the accelerometer off when swimming or bathing/showering to avoid getting the device wet, and during physical contact sports (e.g. rugby) to avoid damaging the device. Data from the returned accelerometers were downloaded and processed in 1-min epochs. After excluding non-wear time (intervals of ≥60 consecutive zero counts), a day with ≥500 wear minutes was considered a valid wear day. An assessment with ≥3 valid wear days^16^ was considered to be a valid assessment. MVPA was defined as ≥2296 ActiGraph vertical counts per minute (cpm) and SED was defined as 0 to <100 cpm.^17^ Because SED time is strongly dependent on monitor wear time^18^ and the percent of SED time per day has been shown to be superior to daily SED time as a predictor of metabolic risk,^19^ daily SED (min/day) was divided by wear time (h/day) to estimate hourly SED (min/h).^20^

To explore a specific type of sedentary behavior, TV viewing, we used self-reported data on time spent watching TV (TV viewing time) collected using the following questions from an unstandardized questionnaire developed for the ALSPAC at ages 13 and 16 years: how much time on average do you spend watching TV each day on a school weekday (or typical weekday). Response options were ‘not at all’, ‘less than 1 hour’, ‘1-2 hours’ and ‘3 or more hours’. This TV viewing measure has been used in previous publications.^21^^,^^22^

### Outcome measurements

The primary outcome was fat mass index (FMI) at age 17 years. During a research clinic visit at age 17 years (between 2008 and 2010), 4851 participants underwent whole-body DXA scans. The scans were performed by trained research staff using a GE Lunar Prodigy narrow-angle fan-beam densitometer. Females who were pregnant (2.8%) were excluded from the DXA scan. DXA scan data were analyzed using in-built GE Lunar enCore software to derive bone mineral content (g), fat mass (kg) and lean mass (kg). FMI was calculated by dividing fat mass by height squared (kg/m^2^). The secondary outcomes were cardiovascular risk factors at age 17 years: systolic blood pressure (mmHg), fasting plasma glucose, triglycerides (mg/dl), high-density lipoprotein cholesterol (mg/dl) and low-density lipoprotein cholesterol (mg/dl). Participants were asked to come to the study clinic in a fasted state. Resting blood pressure was measured twice on each arm using a DINAMAP 9301 Vital Signs Monitor, and the average of the two measures was used to determine blood pressure. A fasting blood sample was drawn, immediately spun and frozen at −80°C. Lipid levels were measured by automated analyzer with enzymatic methods.

### Other variable measurements

To account for socioeconomic effects on adiposity, we considered maternal education at pregnancy <Ordinary (O) level (equivalent to secondary education), O level, or ≥Advanced (A) level] and household income at participant age 9 years (tertiles). Birth weight was also considered^23^ and categorized into two groups: (<2.5 kg or ≥2.5 kg). To account for baseline adiposity, sex- and age-specific BMI percentile at baseline was calculated. Energy intake (calories/day) assessed from a 3-day diet diary at age 13 years^22^ was used as a proxy for daily energy intake in adolescence. Energy intake was categorized into sex-specific tertiles.

### Statistical analyses

Group-based trajectory analyses were conducted to identify distinct trajectory patterns (groups) of SED (min/h) and MVPA (min/day) during adolescence among ALSPAC participants who completed at least two accelerometer assessments. The number of trajectories was determined based on Bayesian Information Criteria. The shape of the trajectories was determined by reducing the level of polynomial functions for each group (i.e. quadratic, linear and constant) until a parameter estimate in the highest function had a significance of *P*<0.01. After fitting the trajectory models, model adequacy was evaluated. More detailed approaches can be found in our prior publication.^24^

The group-based trajectory modeling identified three SED trajectory groups [low (≈25 min/h), middle (≈30 min/h) and high (≈35 min/h)] and four MVPA trajectory groups (Supplementary figure S1). Three MVPA groups with ≥60 min/day of MVPA (‘High1’, ‘High2’ and ‘High3’ in Supplementary figure S1B) were combined as the high MVPA group. Using these two MVPA groups (low and high) and the three SED groups (low, middle and high), a combined MVPA&SED variable for six groups was constructed (High&Low, High&Middle, High&High, Low&Low, Low&Middle and Low&High).

Because TV viewing time was assessed only twice (at age 13 and 16 years), examining its trajectories was not suitable. Instead, we averaged the TV viewing variables (coded 0, 1, 2 and 3 for ‘not at all’, ‘less than 1 hour’, ‘1-2 hours’ and ‘≥3 hours’, respectively) at ages 13 and 16 years among those who had TV viewing data at both time points. The averaged values were divided into three groups: <2 (<1–2 h/day; low), 2 (1–2 h/day; middle) and >2 (>1–2 h/day; high). Using the two MVPA groups and the three TV groups, a combined MVPA&TV variable for six groups was constructed (High&Low, High&Middle, High&High, Low&Low, Low&Middle and Low&High).

For potential confounding variables, missing data were imputed as a middle group (median), as follows. Missing data for maternal education (6% of the sample) were imputed as O level. Missing data for household income (14%) were imputed as a middle tertile group. Missing data for baseline BMI percentile (0.3%) were imputed as 55 (median). Missing data for energy intake (0.2%) were imputed as a middle tertile group.

Pearson correlation coefficients between MVPA, SED and wear time were calculated. Chi-square tests were conducted to compare participant characteristics among the three SED groups (low, middle and high). A multivariable log linear regression model (gamma model with the log link) was built to predict FMI at age 17 years by the six MVPA&SED groups (referent group: High&Low). The model included the following covariates: age, sex and four confounding variables statistically significantly associated with FMI in an age- and sex-adjusted regression model (maternal education, household income, energy intake and baseline BMI percentile). Theses analyses were repeated using the MVPA&TV variable, instead of the MVPA&SED variable. Additional multivariable linear regression analyses were conducted to predict the cardiovascular risk factors.

We conducted stratified analyses separately for those with a high MVPA pattern and those with a low MVPA pattern. As a sensitivity analysis, we conducted sex-specific regression analysis to explore whether the joint associations were similar between males and females. We also examined the associations of the MVPA&SED groups with the outcomes in a subsample who had TV viewing data. A significance level was set at 0.05 (two-sided). All statistical analyses were conducted using SAS 9.4 (Cary, NC).

## Results

Among 3935 participants who completed at least two accelerometer assessments, 402 participants with baseline obesity were excluded from our analysis. Of the remaining 3533 participants, 914 without FMI data at age 17 years were additionally excluded (figure 1). Thus, the main analysis included 2619 participants (56.7% female; 96.5% white; 79.0% with TV viewing data). Compared to the excluded participants, the included participants had a higher proportion of females, white European ancestry and higher maternal education.

The correlation between daily SED (min/day) and daily wear time (min/day) was stronger (*r *=* *0.45; *P *<* *0.01) than the correlation between daily MVPA (min/day) and daily wear time (*r *=* *0.22; *P *<* *0.01). Daily MVPA was negatively correlated with daily SED (*r* = −0.40; *P *<* *0.01) as well as hourly SED (min/h; *r* = −0.56; *P *<* *0.01).

As shown in table 1, 12.3% of the sample was assigned to the low SED pattern, 55.0% to the middle SED pattern and 32.8% to the high SED pattern. For MVPA, 38.7% of the sample was assigned to the high MVPA pattern and 61.3% to the low MVPA pattern. Compared to males, a higher proportion of females followed the high SED pattern (24.0% vs. 39.4%; *P *<* *0.01). TV viewing levels were not associated with SED patterns (*P *=* *0.53).

**Table 1:** The characteristics of participants by three SED trajectory patterns. ALSPAC (*n* = 2619)

	Low SED	Middle SED	High SED
	*n* (%)	*n* (%)	*n* (%)
Total	321 (12.3)	1440 (55.0)	858 (32.8)
Sex^a^			
Male	209 (18.4)	652 (57.6)	272 (24.0)
Female	112 (7.6)	788 (53.0)	586 (39.4)
Birth weight			
<2.5 kg	14 (10.7)	73 (55.7)	44 (33.6)
≥2.5 kg	289 (12.4)	1278 (54.8)	767 (32.9)
Maternal education at pregnancy^a^			
<O level^b^	82 (20.1)	209 (51.2)	117 (28.7)
O level	154 (13.2)	662 (56.8)	350 (30.0)
≥A level	85 (8.1)	569 (54.5)	391 (37.4)
Household income at child age 9 years^a^^,^^c^			
Lowest tertile	88 (8.7)	570 (56.6)	350 (34.7)
Middle tertile	113 (15.5)	392 (53.6)	226 (30.9)
Highest tertile	120 (13.6)	478 (54.3)	282 (32.1)
MVPA trajectory pattern^a^			
High MVPA	252 (24.9)	623 (61.5)	138 (13.6)
Low MVPA	69 (4.3)	817 (50.9)	720 (44.8)
TV viewing time^d^			
Low	72 (10.2)	406 (57.8)	225 (32.0)
Middle	86 (12.0)	381 (53.2)	249 (34.8)
High	73 (11.2)	363 (55.9)	214 (32.9)

	Mean (95% CI)	Mean (95% CI)	Mean (95% CI)

Monitor wear time, min/day			
Age 11 years	781 (775, 788)	786 (783, 789)	785 (781, 789)
Age 13 years	795 (785, 805)	786 (782, 790)	791 (786, 796)
Age 15 years	798 (787, 808)	803 (798, 808)	800 (793, 806)
SED, min/h			
Age 11 years	21 (21, 22)	27 (26, 28)	32 (32, 32)
Age 13 years	24 (24, 25)	30 (30, 31)	37 (37, 37)
Age 15 years	28 (27, 28)	35 (34, 35)	40 (40, 40)
SED, min/day			
Age 11 years	277 (270, 282)	348 (345, 350)	420 (416, 424)
Age 13 years	319 (312, 325)	399 (396, 402)	484 (480, 489)
Age 15 years	368 (359, 378)	463 (459, 468)	533 (528, 539)
MVPA, min/day			
Age 11 years	77 (74, 80)	58 (57, 59)	42 (41, 43)
Age 13 years	76 (73, 80)	54 (53, 56)	39 (38, 41)
Age 15 years	70 (66, 75)	48 (47, 50)	36 (34, 38)

a Chi-square *P*-values <0.05.

b O level is equivalent to secondary education.

c Household income was categorized into tertiles based on the income distribution in the study sample, not in the entire UK population.

d *n* = 2069. 95% CI, 95% confidence interval; MVPA, moderate- and vigorous-intensity physical activity; SED, sedentary time.

Because the absolute values of FMIs differed between males and females, unadjusted mean FMIs are presented separately for males and females (table 2). The mean FMI difference between the High&Low and High&High groups was 0.15 (*P *=* *0.61) among males and 0.24 (*P *=* *0.62) among females. The mean FMI difference between the Low&Low and Low&High groups was 0.56 (*P *=* *0.24) among males and 0.43 (*P *=* *0.34) among females. Unadjusted mean FMIs by MVPA&TV groups are also presented in table 2. The mean FMI difference between the High&Low and High&High groups was 0.55 (*P *<* *0.05) among males and 0.34 (*P *=* *0.31) among females. The mean FMI difference between the Low&Low and Low&High groups was 0.57 (*P *=* *0.07) among males and 0.41 (*P *<* *0.05) among females.

**Table 2:** Unadjusted means of FMI by SED, TV viewing and MVPA groups. ALSPAC (*n* = 2619)

Males		Females	
(*n* = 1133)		(*n* = 1486)	
SED group: mean (95% CI)	MVPA&SED group: mean (95% CI)	SED group: mean (95% CI)	MVPA&SED group: mean (95% CI)
Low: 3.59 (3.29, 3.89)	High&Low: 3.60 (3.27, 3.92)	Low: 6.95 (6.45, 7.45)	High&Low: 6.94 (6.31, 7.57)
	Low&Low: 3.56 (2.79, 4.32)		Low&Low: 6.96 (6.09, 7.84)
Middle: 3.73 (3.55, 3.92)	High&Middle: 3.58 (3.35, 3.80)	Middle: 7.11 (6.94, 7.29)	High&Middle: 6.97 (6.63, 7.30)
	Low&Middle: 3.99 (3.66, 4.32)		Low&Middle: 7.17 (6.96, 7.37)
High: 4.00 (3.70, 4.30)	High&High: 3.75 (3.22, 4.28)	High: 7.38 (7.16, 7.59)	High&High: 7.18 (6.44, 7.91)
	Low&High: 4.12 (3.76, 4.49)		Low&High: 7.39 (7.17, 7.62)
TV group: mean (95% CI)	MVPA&TV group: mean (95% CI)	TV group: mean (95% CI)	MVPA&TV group: mean (95% CI)
Low: 3.45 (3.21, 3.69)	High&Low: 3.33 (3.02, 3.64)	Low: 6.98 (6.75, 7.21)	High&Low: 6.66 (6.20, 7.12)
	Low&Low: 3.60 (3.21, 3.98)		Low&Low: 7.08 (6.81, 7.35)
Middle: 3.69 (3.43, 3.96)	High&Middle: 3.49 (3.19, 3.79)	Middle: 7.14 (6.89, 7.39)	High&Middle: 7.05 (6.48, 7.62)
	Low&Middle: 4.00 (3.52, 4.47)		Low&Middle: 7.16 (6.89, 7.44)
High: 4.00 (3.69, 4.31)	High&High: 3.88 (3.49, 4.28)	High: 7.38 (7.12, 7.63)	High&High: 7.00 (6.52, 7.47)
	Low&High: 4.17 (3.67, 4.67)		Low&High: 7.49 (7.19, 7.79)

95% CI, 95% confidence interval; MVPA, moderate- and vigorous-intensity physical activity; SED, sedentary time; TV, TV viewing time.

**Figure 2:**
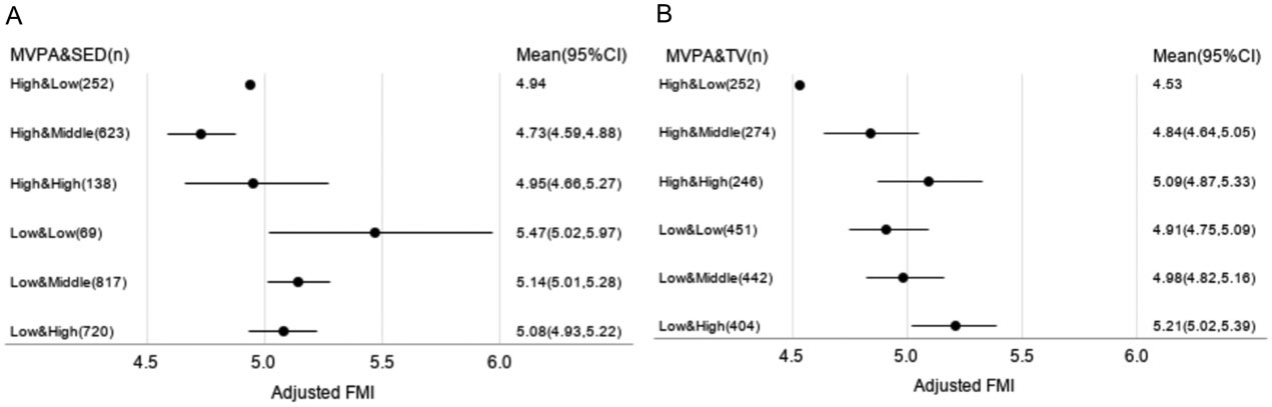
Adjusted means of fat mass index at age 17 years for MVPA&SED and MVPA&TV groups. ALSPAC. A. Adjusted FMI for MVPA&SED groups (n=2,619). B. Adjusted FMI for MVPA&TV groups (n=2,069). Means of FMI were adjusted for age, sex, maternal education, household income, baseline body mass index percentile, and engergy intake. CI, confidence interval; FMI, fat mass index; MVPA, moderate- and vigorous-intensity physical activity; SED, sedentary time; TV, television viewing time.

Because sex was not a modifier in the association of MVPA&SED groups and MVPA&TV groups with FMI (Supplementary tables S1 and S2), we present sex-combined logistic regression analysis results. When FMI was adjusted for the confounding factors, SED patterns were not associated with FMI in combination with either the high or low MVPA pattern (figure 2A). The results remained consistent when the analysis was repeated in a subsample with TV viewing data (*n* = 2069; data not shown). Compared to the high MVPA & low TV group, the other five MVPA&TV groups had significantly higher FMIs (figure 2B). In stratified analysis for those with the high MVPA pattern, higher TV viewing time was associated with higher mean FMI (‘trend’ *P *<* *0.05). Similarly, in stratified analysis for those with the low MVPA pattern, higher SED time was associated with higher FMI (‘trend’ *P *<* *0.05).

Supplementary table S3 presents the associations of MVPA&SED groups and MVPA&TV groups with the cardiovascular risk factor outcomes. Compared to the high MVPA & low SED group, the low MVPA & high SED group had significantly higher triglycerides (mean difference = 4 mg/dl). Overall, there was no significant association of SED or TV viewing with the cardiovascular risk factors examined.

## Discussion

This study found no prospective association between total SED and FMI in either active or inactive participants. However, a distinct sedentary behavior, TV viewing, was prospectively positively associated with FMI in both active and inactive participants. This study found no association of SED or TV viewing with cardiovascular risk factors in active or inactive participants.

The beneficial effect of MVPA on adiposity is strongly supported by epidemiological data^1^ and explained by biological mechanisms.^25^ The link between sedentary behavior and adiposity has also been hypothesized and supported by epidemiologic data in adults.^26^ Potential biological mechanisms for the link have been speculated in animal models: prolonged sitting can cause metabolic alteration, such as suppression of skeletal muscle lipoprotein lipase activity and reduced glucose updake.^27–29^ Among longitudinal studies for the associations of MVPA and SED with adiposity in youth, Kwon et al.^30^ and Janz et al.^31^ reported a significant association of body fat mass with accelerometer-measured MVPA, but not with accelerometer-measured SED, in childhood and adolescence, using the US Iowa Bone Development Study data. Stamatakis et al.^32^ also reported that MVPA at age 11 years, but not SED, was associated with adiposity at age 15 years in the ALSPAC sample. These studies examined the association of MVPA and SED with adiposity by statistically adjusting for each other. However, they did not explicitly test the joint association. Examining the joint association is necessary for understanding how different combinations of PA and sedentary behavior play a role in adiposity development, whereas statistically adjusting for each other examines independent effects. To date, only a few studies have longitudinally examined these joint associations in youth. Among those, Mielke et al.^15^ found no association between self-reported screen time (including TV viewing, videogame play and computer use; >5 h/day vs. ≥5 h/day) at age 11 or 15 years and adiposity at age 18 years among either less active or more active adolescents in the Brazil 1993 Pelotas study sample. Consistent with these prior studies, our findings confirm the result of no association between total SED and adiposity in both active and inactive adolescents. Although the link between total SED and adiposity is biologically plausible, epidemiologic data suggest that the link seems weak in youth after accounting for the MVPA effect. The null-association observed between total SED and adiposity among youth, unlike among adults,^26^ may be partly explained by the youth’s behavioral pattern of a high correlation between MVPA and total SED (*r* = −0.56 in the present sample).

Unlike for total SED, this study found a positive association between TV viewing and adiposity among both active and inactive adolescents. Although TV viewing has often been used as a proxy measure of total SED,^13^ several studies^33–36^ have suggested that specific types of sedentary behavior (e.g. TV viewing, sitting for learning, such as playing an instrument and reading) may have different health effects, which is important in designing successful public health campaigns. Garcia et al.^35^ reported that TV viewing, but not sitting for work, was associated with an increased risk of cardiovascular disease and mortality in adults. TV viewing has been speculated as a unique leisure-time sedentary behavior for adiposity, because beyond low-energy expenditure, it also seems to influence unhealthy diet.^37^ For example, TV viewing is associated with unhealthy eating habits in youth, possibly mediated through TV food advertisements.^37^ Unhealthy eating while watching TV, which is commonly observed,^38^ is also associated with poorer diet quality in youth.^39^ Our findings provide empirical data regarding the uniqueness and specificity of the effect of TV viewing on adiposity and suggest a pathway that is, at least partially, independent of low energy expenditure.

This study revealed that high MVPA did not eliminate nor attenuate the positive association between TV viewing and adiposity. Our findings are somewhat inconsistent with the findings reported in adults showing that high MVPA may eliminate or at least attenuate the increased risk of cardiovascular disease and mortality associated with high TV viewing time.^6^^,^^35^ Although our findings should be replicated in other youth populations, it is plausible that the mechanisms through which TV viewing affect adiposity or other health outcomes (e.g. TV food advertisements) could be different between adults and youth. Future research focused on understanding why TV viewing is a harmful sedentary behavior could help to explain the different results between adults and youth.

The strengths of this study include the use of DXA-derived fat measures, as opposed to BMI, as an indicator of adiposity. Another strength includes the prospective examination of the joint associations, which allowed for establishing a temporal relationship. Additionally, this study used data from multiple accelerometer assessments, which is expected to better represent PA and SED levels across the entire adolescent period, compared to a one-time assessment.

However, several limitations should be acknowledged. First, this study did not examine other screen time, such as time spent on mobile devices, which is now a ubiquitous activity. However, because such devices were not common at the time of data collection, what was being queried (‘watching TV’) would not have been confusing to participants (vs. watching TV shows via a smartphone or watching YouTube videos via TV). In addition, we used the same TV viewing measure that has been used in previous studies, although the validity of that measure has not been reported.^21^^,^^22^ Second, although we excluded participants with obesity at baseline, we cannot rule out that higher FMI could have influenced lower MVPA or higher TV viewing (‘reverse causation’).^40^ However, when we additionally explored the joint associations among adolescents with obesity, we found no significant association (Supplementary figure S2). Third, we cannot rule out the possibility that unmeasured or poorly measured confounders could have contributed to our findings. For example, we did not consider dietary characteristics, such as high-fat diets, which have been shown to be associated with adiposity regardless of PA. Lastly, because this study included only a fraction (<20%) of the entire ALSPAC sample, the results may not represent the entire ALSPAC sample. Furthermore, because the study sample comprised mostly white children, the results may not be generalized to children from other racial/ethnic backgrounds.

In conclusion, this study found that total SED was not associated with adiposity during adolescence among either active or inactive adolescents. However, higher TV viewing time was associated with higher adiposity among both active and inactive adolescents. Our findings suggest that specific types of sedentary behavior could be detrimental to health among youth. This study supports a clearer public health message focusing on increasing MVPA and reducing TV viewing, rather than targeting total SED that includes sitting for learning activities.

## Supplementary data

Supplementary data are available at *EURPUB* online.

## Supplementary Material

ckac023_Supplementary_Data
