# Supplementary material for: Joint associations of physical activity and sedentary time with adiposity during adolescence: ALSPAC
Source: Eur J Public Health. 2022 Apr 13;32(3):347–53. doi: 10.1093/eurpub/ckac023 (PMC9159327; doi:10.1093/eurpub/ckac023)
Supplement: ckac023_Supplementary_Data [file ckac023_supplementary_data.docx]

**Supplementary Data**

Supplementary Table 1. Sex-specific adjusted means (95% confidence intervals) of fat mass index for MVPA&SED and MVPA&TV groups

|  | **Males** | **Females** |
| --- | --- | --- |
| **MVPA&SED** | (n=1,133) | (n=1,486) |
| High&Low | 3.50 (reference) | 7.01 (reference) |
| High&Middle | 3.28 (3.13, 3.43) | 6.89 (6.64, 7.14) |
| High&High | 3.45 (3.14, 3.79) | 7.12 (6.60, 7.67) |
| Low&Low | 3.85 (3.29, 4.50) | 7.68 (7.05, 8.38) |
| Low&Middle | 3.72 (3.52, 3.93) | 7.14 (6.97, 7.30) |
| Low&High | 3.73 (3.49, 3.98) | 7.08 (6.92, 7.25) |
| **MVPA&TV** | (n=845) | (n=1,224) |
| High&Low | 3.13 (reference) | 6.60 (reference) |
| High&Middle | 3.39 (3.17, 3.63) | 7.00 (6.64, 7.39) |
| High&High | 3.60 (3.35, 3.86) | 7.07 (6.69, 7.48) |
| Low&Low | 3.46 (3.20, 3.74) | 6.96 (6.75, 7.18) |
| Low&Middle | 3.54 (3.27, 3.83) | 7.00 (6.80, 7.22) |
| Low&High | 3.80 (3.50, 4.13) | 7.25 (7.03, 7.47) |

Means of fat mass index were adjusted for age, sex, maternal education, household income, baseline body mass index percentile, and energy intake.

MVPA, moderate- and vigorous-intensity physical activity; SED, sedentary time; TV, television viewing time.

Supplementary Table 2. Sex-specific adjusted means (95% confidence intervals) of fat mass for MVPA&SED and MVPA&TV groups

|  | **Males** | **Females** |
| --- | --- | --- |
| **MVPA&SED** | (n=1,133) | (n=1,486) |
| High&Low | 11.1 (reference) | 18.6 (reference) |
| High&Middle | 10.5 (10.1, 11.0) | 19.0 (18.3, 19.7) |
| High&High | 11.1 (10.1, 12.2) | 19.9 (18.4, 21.5) |
| Low&Low | 12.3 (10.5, 14.4) | 20.8 (19.0, 22.7) |
| Low&Middle | 11.8 (11.1, 12.5) | 19.6 (19.2, 20.1) |
| Low&High | 11.9 (11.2, 12.7) | 19.3 (18.8, 19.8) |
| **MVPA&TV** | (n=845) | (n=1,224) |
| High&Low | 10.1 (reference) | 17.9 (reference) |
| High&Middle | 10.8 (10.1, 11.5) | 19.5 (18.4, 20.6) |
| High&High | 11.5 (10.7, 12.3) | 19.4 (18.3, 20.5) |
| Low&Low | 11.1 (10.2, 12.0) | 19.0 (18.4, 19.6) |
| Low&Middle | 11.2 (10.3, 12.1) | 19.2 (18.6, 19.8) |
| Low&High | 12.0 (11.0, 13.0) | 19.8 (19.2, 20.5) |

Means of fat mass index were adjusted for age, sex, maternal education, household income, baseline body mass index percentile, and energy intake.

MVPA, moderate- and vigorous-intensity physical activity; SED, sedentary time; TV, television viewing time.

Supplementary Table 3. Adjusted means (95% confidence intervals) of cardiovascular risk factors for MVPA&SED and MVPA&TV groups

|  | **Systolic blood pressure, mm Hg** | **Fasting glucose,  mg/dL** | **Triglycerides, mg/dL** | **HDL-C, mg/dL** | **LDL-C, mg/dL** |
| --- | --- | --- | --- | --- | --- |
| **MVPA&SED** | (n=2,539) | (n=1,806) | (n=1,806) | (n=1,806) | (n=1,806) |
| High&Low | 117 (reference) | 91 (reference) | 69 (reference) | 50 (reference) | 81 (reference) |
| High&Middle | 117 (116, 118) | 91 (90, 91) | 71 (68, 74) | 50 (49, 51) | 79 (77, 81) |
| High&High | 115 (114, 117) | 92 (90, 94) | 72 (66, 78) | 51 (49, 53) | 79 (74, 84) |
| Low&Low | 119 (117, 121) | 90 (88, 92) | 76 (68, 85) | 47 (44, 50) | 78 (72, 84) |
| Low&Middle | 116 (116, 117) | 90 (89, 91) | 72 (69, 75) | 49 (48, 50) | 84 (82, 86) |
| Low&High | 116 (115, 117) | 91 (90, 92) | 73 (70, 76) | 49 (48, 50) | 81 (79, 83) |
| **MVPA&TV** | (n=2,017) | (n=1,432) | (n=1,432) | (n=1,432) | (n=1,432) |
| High&Low | 117 (reference) | 90 (reference) | 72 (reference) | 50 (reference) | 81 (reference) |
| High&Middle | 117 (116, 118) | 91 (90, 93) | 72 (68, 77) | 50 (48, 51) | 81 (77, 84) |
| High&High | 117 (116, 118) | 91 (90, 93) | 70 (65, 74) | 50 (49, 52) | 78 (75, 82) |
| Low&Low | 117 (116, 117) | 90 (89, 91) | 72 (68, 75) | 50 (49, 51) | 81 (79, 84) |
| Low&Middle | 116 (115, 117) | 91 (90, 92) | 72 (68, 75) | 49 (47, 50) | 84 (81, 86) |
| Low&High | 116 (116, 117) | 90 (89, 91) | 75 (71, 79) | 48 (47, 49) | 83 (80, 86) |

Means of cardiovascular risk factors were adjusted for age, sex, maternal education, household income, baseline body mass index percentile, and energy intake.

HDL-C, high-density lipoprotein cholesterol; LDL-C, low-density lipoprotein cholesterol; MVPA, moderate- and vigorous-intensity physical activity; SED, sedentary time; TV, television viewing time.

Supplementary Figure 1. Trajectory patterns of sedentary time and moderate-and vigorous-intensity physical activity between age 11 and 15 years. ALSPAC (n=3,935).

1. Sedentary time trajectories


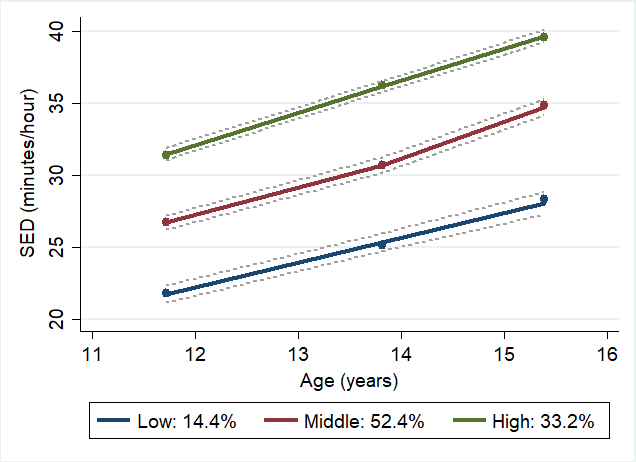


1. Moderate- and vigorous-intensity physical activity trajectories


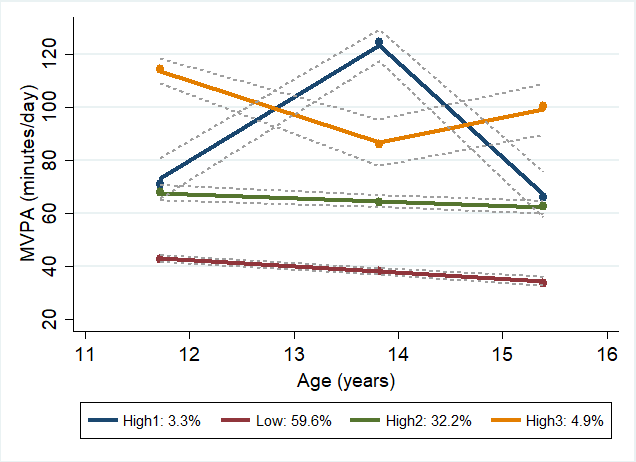


Dots are calculated means at each assessment. Solid lines are estimated values from trajectory models and dotted lines are 95% confidence intervals. MVPA, moderate- and vigorous-intensity physical activity; SED, sedentary time.

Supplementary Figure 2. Adjusted means of fat mass index for MVPA&SED and MVPA&TV groups among ALSPAC participants with obesity at age 11 years.

1. Adjusted FMI for MVPA&SED groups (n=267)


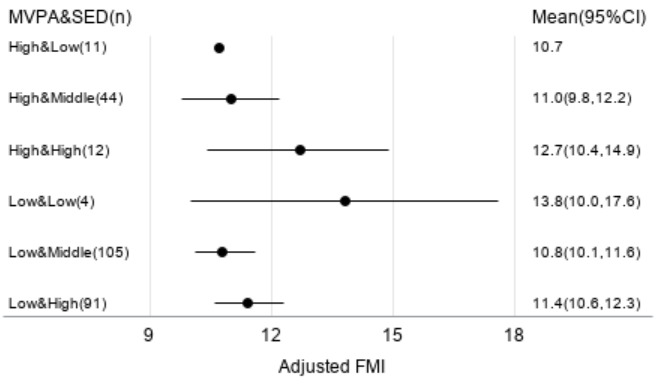


1. Adjusted FMI for MVPA&TV groups (n=212)


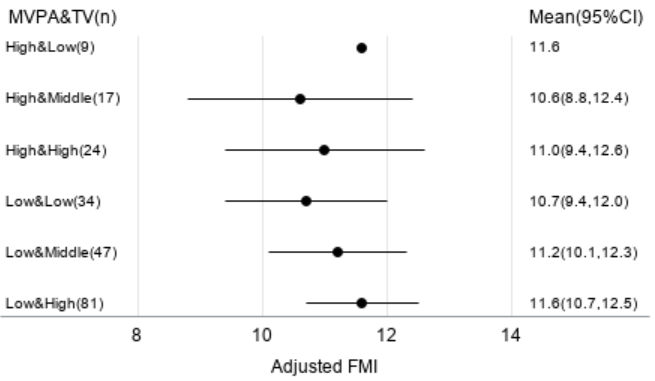


Note: Means of FMI were adjusted for age, sex, maternal education, household income, baseline body mass index percentile, and energy intake. CI, confidence interval; FMI, fat mass index; MVPA, moderate- and vigorous-intensity physical activity; SED, sedentary time; TV, television viewing time.
